# Formyl peptide receptors promotes neural differentiation in mouse neural stem cells by ROS generation and regulation of PI3K-AKT signaling

**DOI:** 10.1038/s41598-017-00314-5

**Published:** 2017-03-16

**Authors:** Liang Zhang, Guan Wang, Xingxing Chen, Xin Xue, Qiaonan Guo, Mingyong Liu, Jianhua Zhao

**Affiliations:** 1 Department of Spine Surgery, Daping Hospital, Third Military Medical University, Chongqing, 400042 China; 2 Department of Pathology, Xinqiao Hospital, Third Military Medical University, Chongqing, 400037 China

## Abstract

This study aimed to determine whether formyl peptide receptors (FPRs) regulated the differentiation of neural stem cells (NSCs). FPRs promote the migration of NSCs both *in vitro* and *in vivo*. However, the role of FPRs during differentiation of NSCs is unknown. Analysis by Western blot showed significantly increased expression of FPR1 and FPR2 during differentiation of NSCs. The activation of FPRs promotes NSCs to differentiate into neurons with more primary neurites and branch points and longer neurites per cell. Meanwhile, this activation also inhibits the differentiation of NSC into astrocytes. This bidirectional effect can be inhibited by the FPRs-specific inhibitor. Moreover, it was found that the activation of FPRs increased the generation of reactive oxygen species (ROS) and phosphorylation of AKT in the NSCs, while *N*-acetylcysteine and LY294002 inhibited the FPRs-stimulated increase in ROS generation and AKT phosphorylation, and blocked the FPRs-stimulated neural differentiation into neurons. Therefore, FPRs-stimulated neural differentiation was mediated via ROS and PI3K**-AKT** signaling pathways. Collectively, the present findings provided a novel insight into the functional role of FPRs in neurogenesis, with important implications for its potential use as a candidate for treating brain or spinal cord injury.

## Introduction

Neural stem cells (NSCs) are self-renewing cells capable of differentiating into neurons, astrocytes, and oligodendrocytes^1, 2^. Recently, numerous studies have confirmed the contribution of NSCs to the recovery of nerve function following spinal cord injury^3–7^. NSCs are used to repair the damaged nervous system, either by transplantation of cells grown *in vitro* or by activation of endogenous stem cells. Their targeted migration toward injured areas and specific differentiation into functional neurons are the key to success. Most NSCs differentiate into neuroglial cells in the injured areas^8, 9^. Therefore, promoting the capability of NSCs of differentiating into neurons is crucial to their potential contribution to the regeneration of damaged central nervous system (CNS).

Formyl peptide receptors (FPRs) are members of the G protein−coupled receptors (GPCRs). FPR has three isoforms: FPR1, FPR2, and the human-exclusive FPR3^10–13^. Expression of FPR in phagocytic leukocytes has been reported^14–16^. FPRs are chemical chemokine receptors that can bind multiple *N*-formyl peptides such as *N*-formyl-methionyl-leucyl-phenylalanine (fMLP) and Annexin A1^17–19^. Meanwhile, ligands for FPRs were found during inflammatory processes such as bacterial infection and skin injury^20, 21^. After binding to their ligands, FPRs trigger multiple signaling pathways such as extracellular signal−regulated kinase 1/2 and JUNK, promoting the migration of inflammatory cells, enhancing the phagocytic capacity, and increasing the gene transcription and release of reactive oxygen species (ROS) and nitric oxide (NO)^15^. Extensive studies have been conducted in recent years to investigate the expression and effect of FPRs in nonphagocytic cells such as fibroblasts^22^. It is confirmed that mesenchymal stem cells (MSCs) also express FPRs, and FPRs promote the migration of MSCs and induce their differentiation^23–25^. Increasing evidence indicate that FPRs, including FPR1 and/or FPR2, expressed in the CNS have the ability to interact with formyl-methyl-leucyl-phenylalanine (fMLF/fMLP)^26, 27^. These receptors have been detected in human brain, spinal cord, anterior horn cells, and hypoglossal nucleus neurons^28^.

As shown by previous data, FPRs were also expressed in NSCs and promoted the migration of rat NSCs both *in vitro* and *in vivo* ^29^. The present study mainly discussed the roles and mechanisms of FPRs in promoting neuronal differentiation of NSCs. This study showed significantly increased expression of FPR1 and FPR2, during differentiation of NSCs. The activation of FPRs promotes NSCs to differentiate into neurons with more primary neurites and branch points and longer neurites per cell. Meanwhile, this activation also inhibits the differentiation of NSCs into astrocytes. Moreover, it was also found that FPRs-stimulated neural differentiation was mediated via ROS and PI3K-AKT signaling pathways.

## Results

### Expression of FPRs significantly increased during differentiation of mouse NSCs

**Figure 1:**
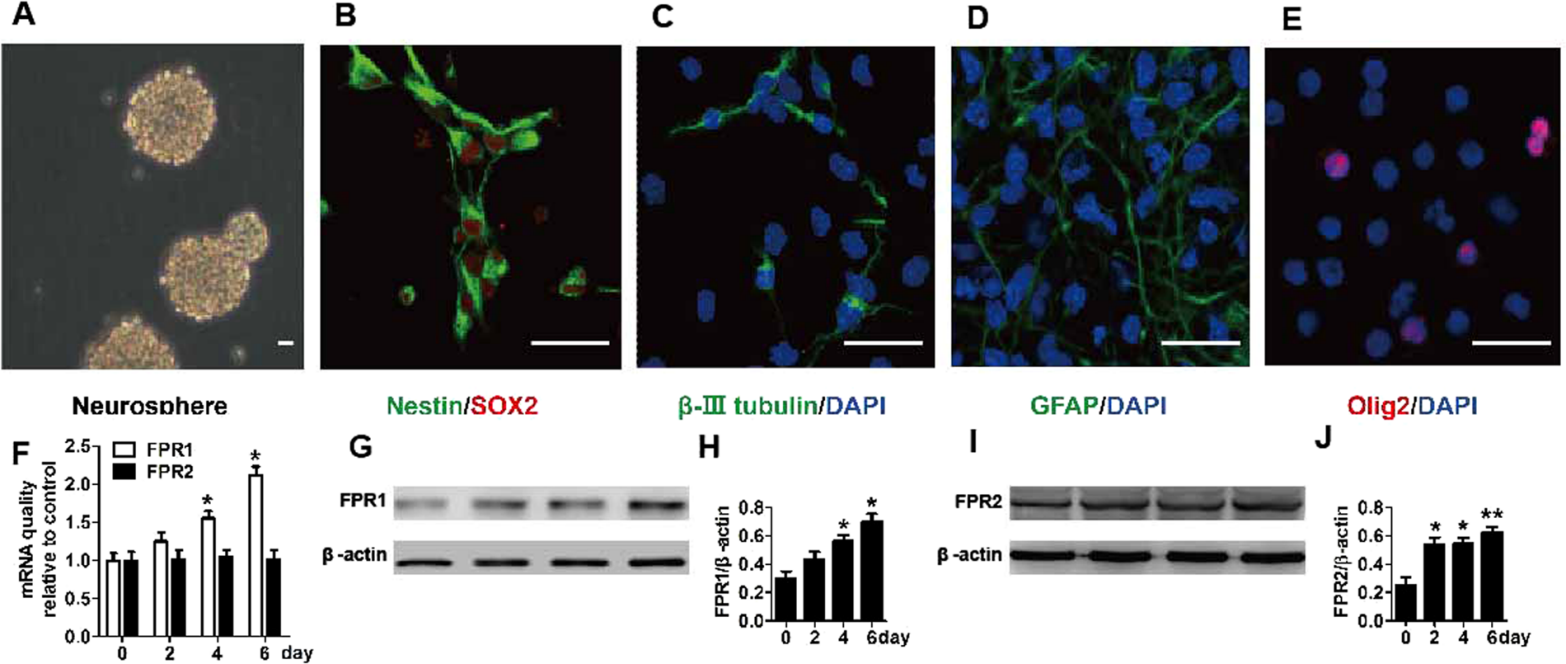
Characterization of neurospheres from cortices of fetal mouse pups and expression of FPRs significantly increasing during differentiation of mouse NSCs. (**A**) Phase contrast images of suspended neurospheres. (**B–E**) Confocal images of adherent cells. (**B**) NSCs labeled with SOX2 and Nestin. (**C–E**) Neurons, astrocytes, and oligodendrocytes. (**C**) β-III tubulin−labeled neurons with DAPI-counterstained nuclei. (**D**) GFAP-labeled astrocytes with DAPI-counterstained nuclei. (**E**) Olig2-labeled oligodendrocytes with DAPI-counterstained nuclei. (**F**) FPR1 and FPR2 mRNA expression during differentiation of mouse NSCs was evaluated by quantitative reverse transcription PCR. (**G**) Representative immunoblots for FPR1. (**H**) Histograms showing relative levels of FPR1. (**I**) Representative immunoblots for FPR2. (**J**) Histograms showing relative levels of FPR2. Data were presented as means ± SEM. *P < 0.05; *N* = 6. Scale bar = 20 µm.

Cortical cells of fetal E12.5 mouse pups were plated in a culture dish in the DMEM/F12 medium supplemented with B27, 20 ng/mL EGF, and 20 ng/mL FGF-2 at 37 °C under humidified 5% CO_2_ conditions. After 7-day culture, a typical neurosphere would appear (Fig. 1A). After digestion with StemProAccutase Cell Dissociation Reagent, the cells were seeded in the polyornithine-coated dishes. The cells then adhered to the wall and grew with extending neurites. Under these growth conditions, virtually all cells expressed Sox-2, a marker of early neural progenitors. Nestin, another marker of NSCs, was also expressed in most cells (Fig. 1B). To further assess the differentiation potential of the obtained NSCs, differentiation was induced, revealing that NSCs could differentiate into three types of cells, that expressed beta-III tubulin, GFAP, and Olig2, respectively. This result indicated that cultured NSCs were capable of differentiating into neurons, astrocytes, and oligodendrocytes (Fig. 1C–E).

To determine whether expression of FPR family members was regulated during differentiation of mouse NSCs, the NSCs were differentiated in the DMEM/F12 medium supplemented with 1% FBS and FPR expression was analyzed. As shown in Fig. 1, expression of FPR1 mRNA, but not FPR2 mRNA, significantly increased in a time-dependent manner during differentiation of NSCs, as analyzed by real-time PCR (Fig. 1F). The expression of FPR1 protein analyzed by Western blot was in line with the result of real-time PCR (Fig. 1G and H). Meanwhile we observed that the expression of FPR2 protein significantly increased in a time-dependent manner during differentiation of NSCs analyzed by Western blot (Fig. 1I and J).

### FPRs promoted neuronal differentiation of NSCs

To determine whether FPRs in NSCs was functional during neuronal differentiation, the FPRs agonists were used in varying concentrations to stimulate the cells and observe changes in their differentiation potentials.

**Figure 2:**
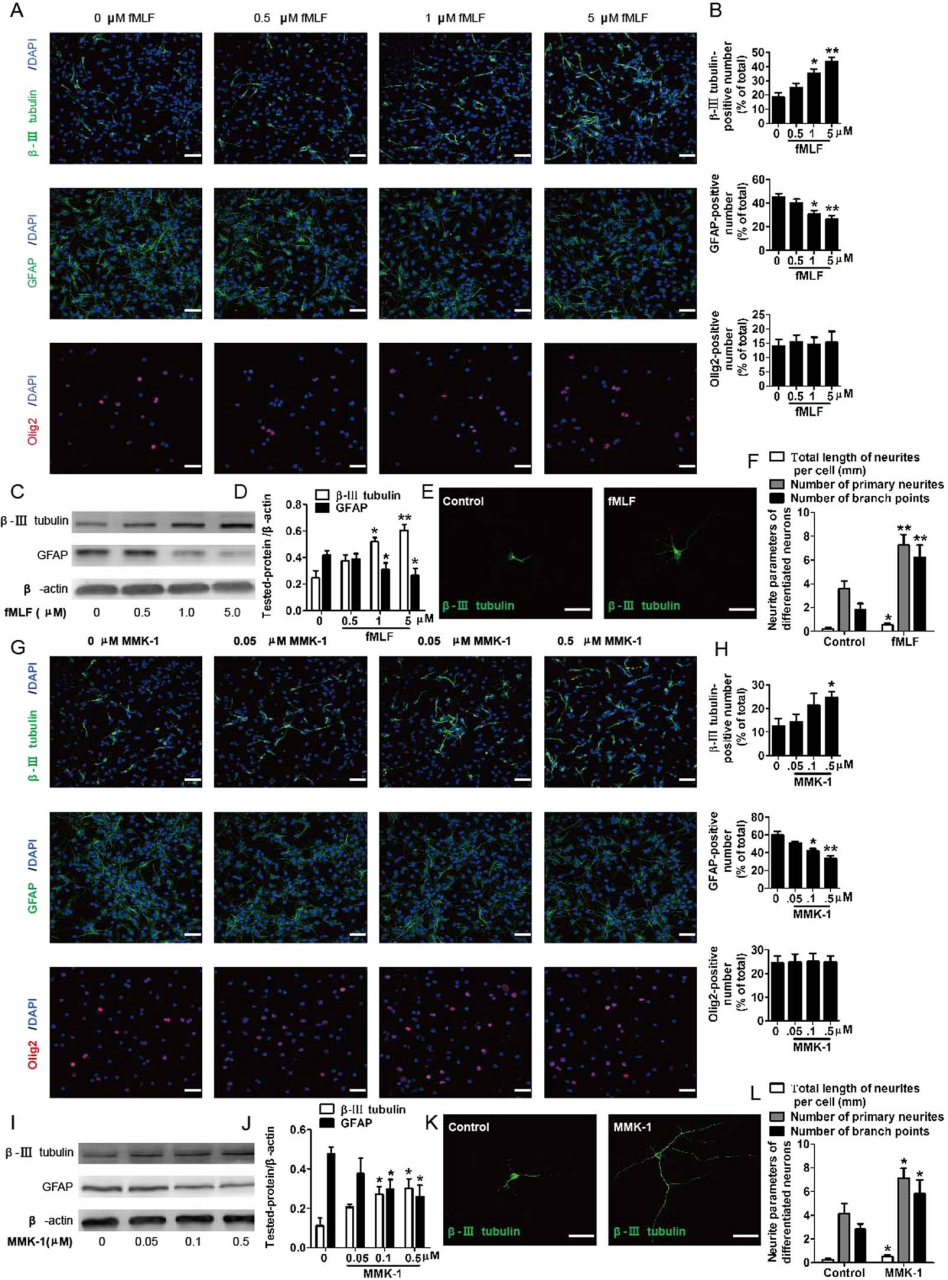
FPRs promoted neuronal differentiation of NSCs. (**A–F**) FPR1 promoted neuronal differentiation of NSCs. (**A,B**) Neurospheres were dissociated into single cells and cultured in a differentiation medium with 0, 0.5, 1, and 5 µM fMLF for 6 days. The differentiated cells were then identified by immunocytochemistry using antibodies against β-III tubulin, GFAP, and Olig2. The images in (**A**) are representative of several independent experiments, and the graph in (**B**) shows the statistical results. (**C**) Representative immunoblots. (**D**) Histograms showing relative levels of β-III tubulin and GFAP. (**E**) Dissociated NSCs were differentiated in an extremely low density (500 cells/cm^2^), and then the cells were immunostained with β-III tubulin. (**F**) Quantified analysis of the total length of neurites per cell, the number of primary neurites, and the number of branch points. (**G–L**) FPR2 promoted neuronal differentiation of NSCs. (**G,H**) Neurospheres were dissociated into single cells and cultured in a differentiation medium with 0, 0.05, 0.1, and 0.5 µM MMK-1 for 6 days. The images in (**G**) are representative of several independent experiments, and the graph in (**H**) shows the statistical results. (**I**) Representative immunoblots. (**J**) Histograms showing relative levels of β-III tubulin and GFAP. (**K**) Representative micrographs of the differentiated cells immunostained with β-III tubulin. (**L**) Quantified analysis of the total length of neurites per cell, the number of primary neurites, and the number of branch points. Data were presented as mean ± SEM. *P < 0.05; **P < 0.01; *N* = 4. Scale bar = 20 µm.

The effects of FPRs on NSC differentiation were investigated by immunostaining using the neuron-specific marker β-III tubulin, the astrocyte-specific marker GFAP, and the oligodendrocyte-specific marker Olig2. The results of confocal microscopy assay indicated that fMLF (FPR1 agonist) or MMK-1 (FPR2 agonist) had concentration-dependent promoting effect on NSC differentiation into neurons, as revealed by significantly increased levels of neuron marker β-III tubulin. Meanwhile, as concentrations of fMLF or MMK-1 increased, the percentage of glial fibrillary acidic protein (GFAP)-positive cells gradually decreased while the percentage of Olig2-positive cells remained unchanged (Fig. 2A,B,G and H). These results suggested that FPR1 or FPR2 promoted the differentiation of NSCs into neurons and inhibited the differentiation into astrocytes. In addition, the expression of β-III tubulin and GFAP was examined by Western blot. The results showed that the β-III tubulin levels were robustly upregulated, while the GFAP levels were significantly downregulated after fMLF or MMK-1 treatment (Fig. 2C,D,I and J). Furthermore, fMLF or MMK-1 increased the number of β-III tubulin−positive neurites as detected by inducing single NSC differentiation for 6 days, and the fMLF or MMK-1-induced differentiated neurons appeared with more branch points and longer neurites (Fig. 2E,F,K and L).

To further confirm the promoting effect of FPRs on the differentiation of NSCs into neurons, its inhibitory effect on the differentiation of NSCs into astrocytes, and its effect on increasing the length of neurites and number of primary neurites and branch points in newly differentiated neurons, the FPRs-specific antagonists were used to carry out corresponding blocking experiments.

**Figure 3:**
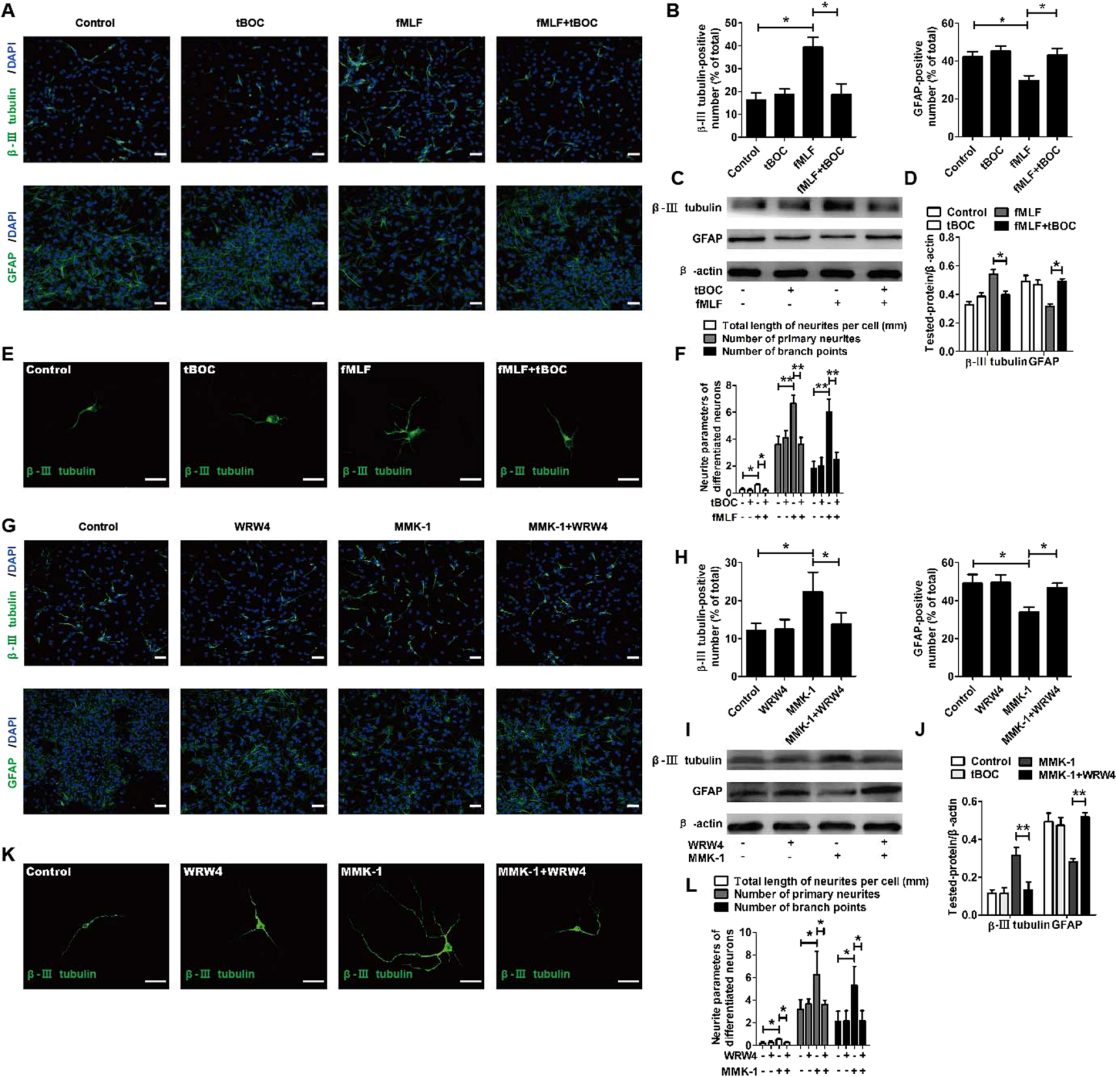
Promoting effects of Fprs on neuronal differentiation of NSCs were blocked by FPRs antagonist. (**A–F**) Promoting effects of Fpr1 on neuronal differentiation of NSCs were blocked by FPR1 antagonist. (**A,B**) Dissociated NSCs were differentiated for 6 days with or without preadministering tBOC, an FPR1 inhibitor, for 30 min before administering fMLF. The differentiated cells were then identified by immunocytochemistry using antibodies against β-III tubulin and GFAP. The images in (**A**) are representative of several independent experiments, and the graph in (**B**) shows the statistical results. (**C**) Representative immunoblots. (**D**) Histograms showing relative levels of β-III tubulin and GFAP. (**E**) Dissociated NSCs were differentiated in an extremely low density (1000 cells/mL), and then the cells were immunostained with β-III tubulin. (**F**) Quantified analysis of total length of neurites per cell, the number of primary neurites, and the number of branch points. (**G–L**) Promoting effects of Fpr2 on neuronal differentiation of NSCs were blocked by FPR2 antagonist. (**G,H**) Dissociated NSCs were differentiated for 6 days with or without preadministering WRW4, an FPR2 inhibitor, for 30 min before administering MMK-1. The images in (**G**) are representative of several independent experiments, and the graph in (**H**) shows the statistical results. (**I**) Representative immunoblots. (**J**) Histograms showing relative levels of β-III tubulin and GFAP. (**K**) Representative micrographs of the differentiated cells immunostained with β-III tubulin. (**L**) Quantified analysis of total length of neurites per cell, the number of primary neurites, and the number of branch points. Data were presented as mean ± SEM. *P < 0.05; **P < 0.01; *N* = 4. Scale bar = 20 µm.

The results of confocal microscopy indicated that a significant increase in the percentage of β-III tubulin-positive cells and a significant decrease in the percentage of GFAP-positive cells induced by fMLF or MMK-1 were abolished by the tBOC (FPR1 agonist) or WRW4 (FPR2 agonist) (Fig. 3A,B,G and H). In addition, the expression of β-III tubulin and GFAP was examined by Western blot. The results showed that a significant increase in β-III tubulin levels and a significant decrease in GFAP levels induced by fMLF or MMK-1 were abolished by tBOC or WRW4 (Fig. 3C,D,I and J). Notably, tBOC or WRW4 also eliminated the effects of fMLF or MMK-1 on the neurite outgrowth, the number of primary neurites, and the number of branch points of β-III tubulin-positive cells (Fig. 3E,F,K and L). The results showed that the effects of fMLF or MMK-1 were blocked by FPRs antagonists.

### FPRs is expressed in neurosphere-dissociated cells

**Figure 4:**
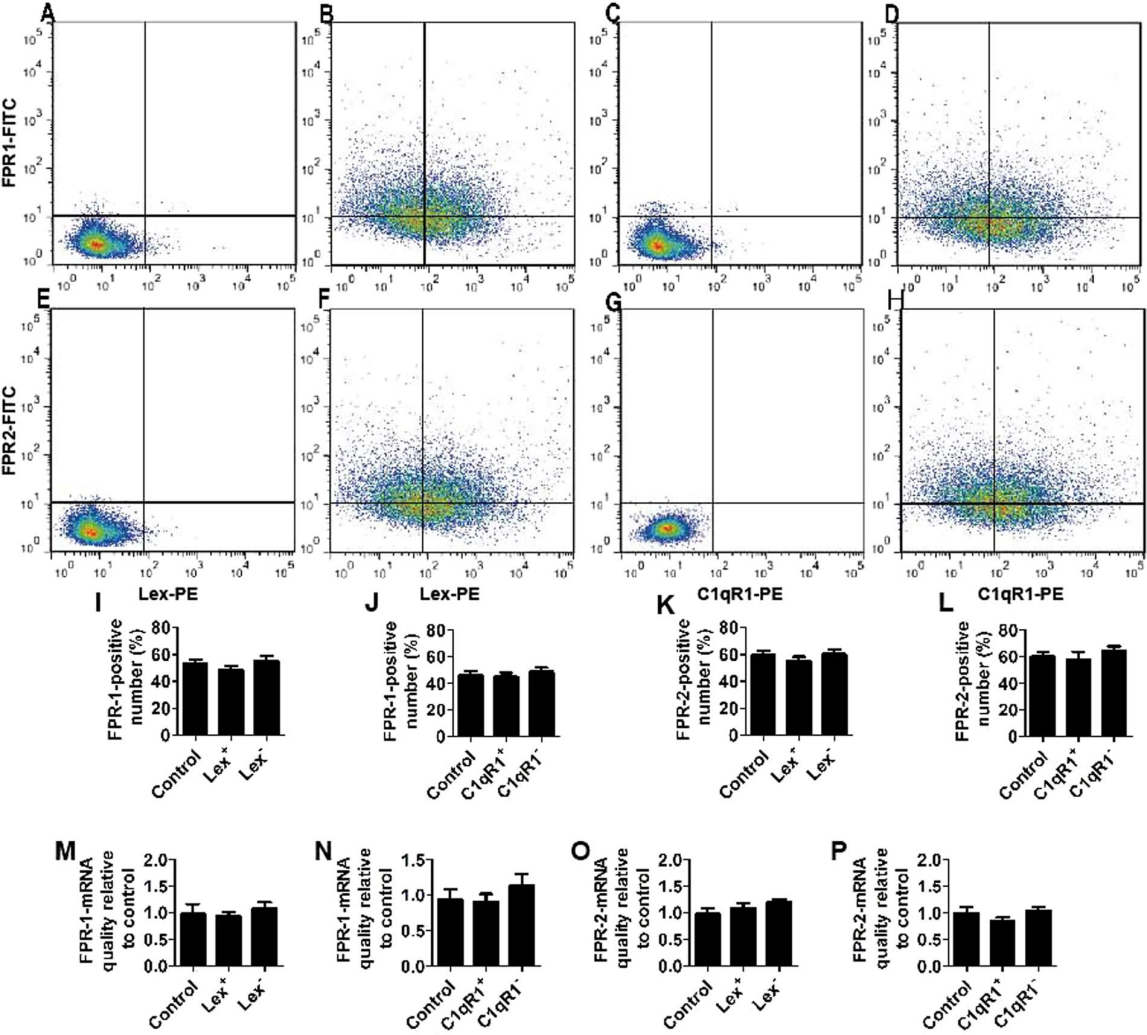
FPRs is expressed in neurosphere-dissociated cells. (**A–L**) Flow cytometric analyses of expression of FPR1 and FPR2 at the protein level. Neurosphere-dissociated cells doubly stained with C1qR1 or LeX and FPR1 or FPR2 antibody were assayed by flow cytometry. Results are representative of at least three independent experiments. (**A**) Negative control for LeX/FPR1, (**B**) LeX/FPR1, (**C**) negative control for C1qR1/FPR1, (**D**) C1qR1/FPR1. (**E**) Negative control for LeX/FPR2, (**F**) LeX/FPR2, (**G**) negative control for C1qR1/FPR2, (**H**) C1qR1/FPR2. (**I**) Histograms showing the percentages of FPR1 positive cells in total, Lex^+^, and Lex^−^ cells, (**J**)FPR1 positive cells in total, C1qR1^+^, and C1qR1^−^ cells, (**K**) FPR2 positive cells in total, Lex^+^, and Lex^−^ cells, (**L**) FPR2 positive cells in total, C1qR1^+^, and C1qR1^−^ cells. (**M–P**) FPR1 and FPR2 mRNA expression of LeX/C1qR1-sorted cells from dissociated neurospheres was evaluated by quantitative reverse transcription PCR. Results are representative of at least three independent experiments. Data were presented as mean ± SEM; *P < 0.05; N = 6.

Neurospheres themselves are not a pure population of NSCs; they comprise a heterogeneous mix of cells, which includes lineage-restricted NPs^30, 31^. To further identify which cell type in neurosphere expresses FPRs, in this work, neurosphere-dissociated cells doubly stained with C1qR1 or LeX and FPR1 or FPR2 antibody were assayed by flow cytometry. The most widely used markers for NSCs is Lewis-X (LeX)^32^. Recently Yu identified C1qR1 as a novel marker for NSCs^33^. The results of flow cytometry suggested that FPR1 and FPR2 were partially expressed in Lex^+^ and Lex^−^ cells or C1qR1^+^ and C1qR1^−^ cells (Fig. 4A–L). Meanwhile FPR1 and FPR2 expression for LEX-positive or C1qR1-positive cells were not significantly higher than that for negative cells. To further identify this result, neurosphere-dissociated cells stained with C1qR1 or LeX antibody were sorted using a Becton Dickinson FACS Aria. FPR1 and FPR2 mRNA expression of LeX or C1qR1-sorted cells from dissociated neurospheres was evaluated by quantitative reverse transcription PCR. As shown in Fig. 4M–P, expression of FPR1 and FPR2 mRNA, for LEX-positive or C1qR1-positive cells were not significantly higher than that for negative cells. These results suggested that FPR1 and FPR2 were partially expressed in neurosphere-dissociated cells (including NSCs, non-neural stem cells for example: lineage-restricted NPs) and FPR1 and FPR2 expressed in NSCs were not different from that for non-neural stem cells.

### FPRs promoted neuronal differentiation of Lex^+^/C1qR1^+^ cells from dissociated neurospheres

**Figure 5:**
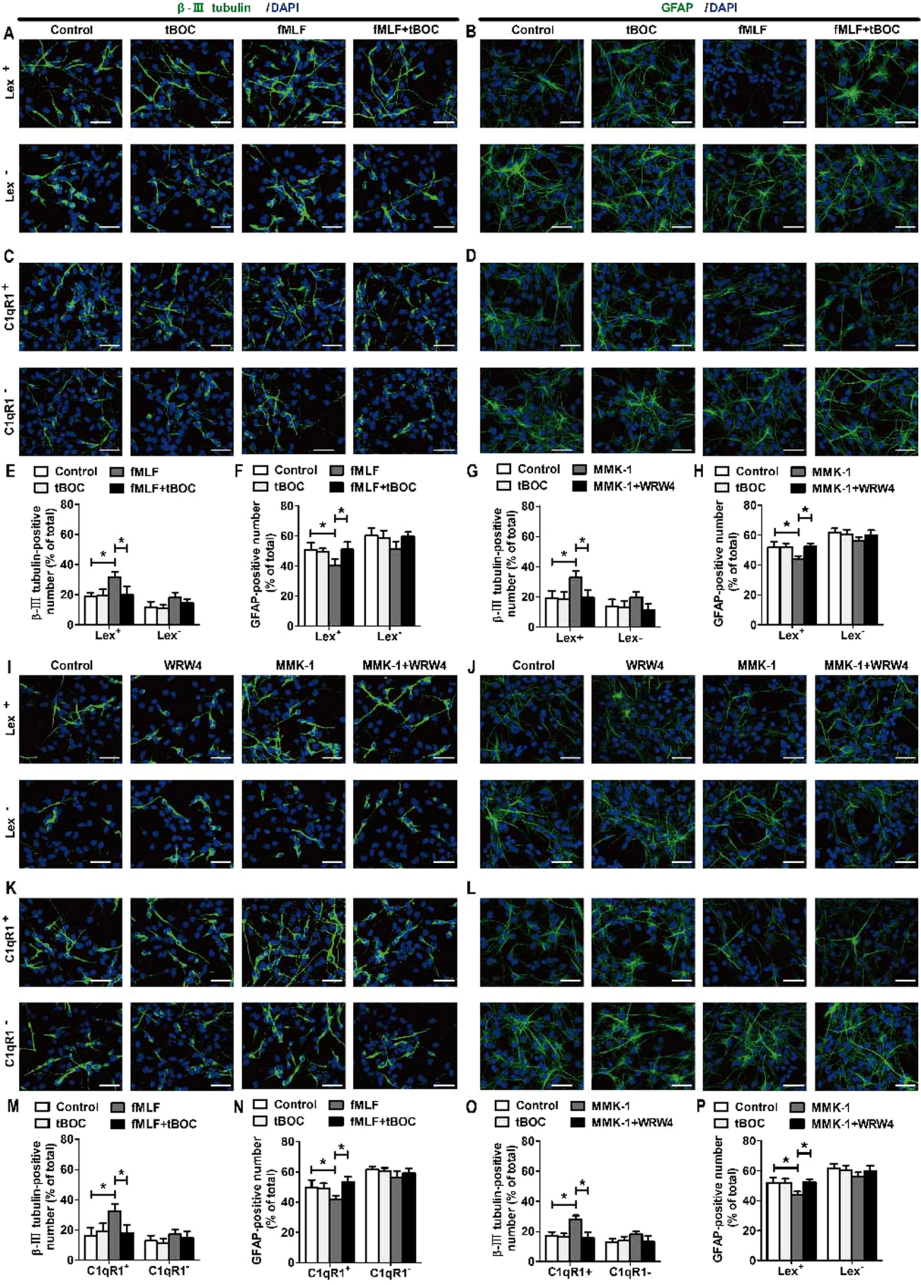
FPRs promoted neuronal differentiation of Lex^+^ or C1qR1^+^ cells from dissociated neurospheres. (**A**–**H**) FPR1 promoted neuronal differentiation of Lex^+^ or C1qR1^+^ cells. Cells were sorted with markers, including Lex or C1qR1. Each cell population was cultured for 4 days with or without preadministering tBOC for 30 min before administering fMLF, before proceeding to culture with neural differentiation medium. The differentiated cells were then identified by immunocytochemistry using antibodies against β-III tubulin and GFAP. The images in (**A–D**) are representative of several independent experiments, and the graph in (**E**–**H**) shows the statistical results. (**I–P**) FPR2 promoted neuronal differentiation of Lex^+^ or C1qR1^+^ cells. Each cell population was cultured for 4 days with or without preadministering WRW4 for 30 min before administering MMK-1, following to culture with neural differentiation medium. The differentiated cells were then identified by immunocytochemistry using antibodies against β-III tubulin and GFAP. The images in (**I–L**) are representative of several independent experiments, and the graph in (**M–P**) shows the statistical results. Data were presented as mean ± SEM; *P < 0.05. N = 6. Scale bar = 20 µm.

**Figure 6:**
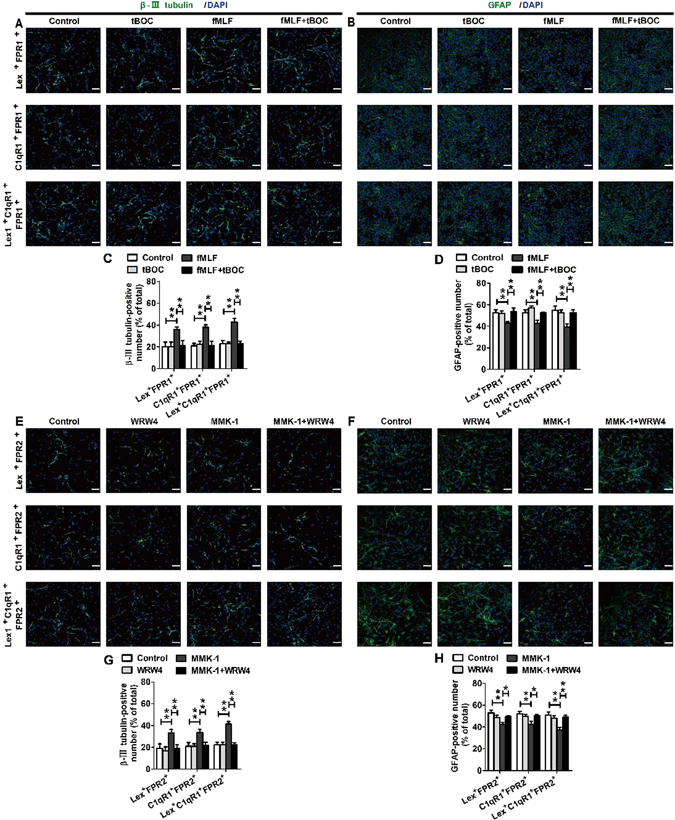
FPRs promoted neuronal differentiation of Lex^+^FPR^+^, C1qR1^+^FPR^+^ or Lex^+^C1qR1^+^FPR^+^ cells from dissociated neurospheres. (**A–D**) FPR1 promoted neuronal differentiation of Lex^+^FPR1^+^, C1qR1^+^FPR1^+^ or Lex^+^C1qR1^+^FPR1^+^ cells. Cells were sorted using a Becton Dickinson FACS Aria with markers, including Lex, C1qR1 or FPR1. Each cell population was cultured for 4 days with or without preadministering tBOC for 30 min before administering fMLF, before proceeding to culture with neural differentiation medium. The differentiated cells were then identified by immunocytochemistry using antibodies against β-III tubulin and GFAP. The images in (**A,B**) are representative of several independent experiments, and the graph in (**C,D**) shows the statistical results. (**E–H**) FPR2 promoted neuronal differentiation of Lex^+^FPR2^+^, C1qR1^+^FPR2^+^ or Lex^+^C1qR1^+^FPR2^+^ cells. Each cell population was cultured for 4 days with or without preadministering WRW4 for 30 min before administering MMK-1, following to culture with neural differentiation medium. The differentiated cells were then identified by immunocytochemistry using antibodies against β-III tubulin and GFAP. The images in (**E,F**) are representative of several independent experiments, and the graph in (**G,H**) shows the statistical results. Data were presented as mean ± SEM; *P < 0.05. N = 6. Scale bar = 20 µm.

The results of flow cytometry indicate that FPR1 and FPR2 were expressed in NSCs. To further identify FPR1 and FPR2 were functional in neural differentiation of NSCs, neurosphere-dissociated cells stained with C1qR1 or LeX antibody were sorted using a Becton Dickinson FACS Aria. Each cell population was first treated with fMLF or MMK-1 for 7 days (for the blocked group, tBOC or WRW4 was added 30 min prior to the treatment), and then the culture media was replaced with the differentiation media (no longer containing FPR agonists or antagonists). After 6 days of differentiation, immunofluorescence staining using β-III tubulin and GFAP was performed, and the results were observed using the confocal microscopy assay. The specific groups were as follows: control group, tBOC or WRW4 group, fMLF or MMK-1 group, and fMLF + BOC or MMK-1 + WRW4 group, for both Lex^+^ and C1qR1^+^ cells and Lex^−^ and C1qR1^−^ cells. The confocal results showed that the fMLF group for Lex^+^ or C1qR1^+^ cells could produce more β-III tubulin–positive cells and fewer GFAP-positive astrocytes, while the fMLF + BOC group for Lex^+^ or C1qR1^+^ cells contained the number of neurons and astrocytes restoring to the level of the control group. In the fMLF group for Lex^−^ and C1qR1^−^ cells, a trend of production of more β-III tubulin–positive cells and fewer GFAP-positive astrocytes was observed (Fig. 5A–H). The MMK-1 group for Lex^+^ or C1qR1^+^ cells could produce more β-III tubulin–positive cells and fewer GFAP-positive astrocytes, while the MMK-1 + WRW4 group for Lex^+^ or C1qR1^+^ cells contained the number of neurons and astrocytes restoring to the level of the control group. In the MMK-1 group for Lex^−^ and C1qR1^−^ cells, only a trend of production of more β-III tubulin–positive cells and fewer GFAP-positive astrocytes was observed (Fig. 5I–P). To further identify FPRs were functional in neural differentiation of NSCs, neurosphere-dissociated cells doubly or triply stained with C1qR1, LeX or FPRs antibody were sorted using a Becton Dickinson FACS Aria. Other treatments were the same as above. The confocal results showed that the fMLF group for Lex^+^FPR1^+^, C1qR1^+^FPR1^+^ or Lex^+^C1qR1^+^FPR1^+^ cells could produce more β-III tubulin–positive cells and fewer GFAP-positive astrocytes, while the fMLF + BOC group for Lex^+^FPR1^+^, C1qR1^+^FPR1^+^ or Lex^+^C1qR1^+^FPR1^+^ cells contained the number of neurons and astrocytes restoring to the level of the control group (Fig. 6A–D). The MMK-1 group for Lex^+^FPR2^+^, C1qR1^+^FPR2^+^ or Lex^+^C1qR1^+^FPR2^+^ cells could produce more β-III tubulin–positive cells and fewer GFAP-positive astrocytes, while the MMK-1 + WRW4 group for Lex^+^FPR1^+^, C1qR1^+^FPR1^+^ or Lex^+^C1qR1^+^FPR1^+^ cells contained the number of neurons and astrocytes restoring to the level of the control group (Fig. 6E–H). These results indicated that the activation of FPR1 and FPR2 promoted the neural differentiation and inhibited the differentiation of Lex^+^, C1qR1^+^ or Lex^+^C1qR1^+^ cells into astrocytes.

### FPRs-induced neuronal differentiation of NSCs was mediated by ROS generation

FPRs were first reported in the studies on phagocytic leukocytes and proved to promote the phagocytic capacity and ROS generation of leukocytes^34–36^. Since ROS is a signaling molecule involved in the process during which neurotrophic factors promote the differentiation of NSCs into neurons^37–40^, the question is whether ROS is also a signaling molecule participating in the process during which FPRs strengthens NSCs’ potential to differentiate into neurons. The corresponding experiments were performed: the level of intracellular ROS was measured using flow cytometry; neuronal differentiation (percentage of β-III tubulin cells) and astrocyte differentiation (percentage of GFAP cells) were examined by confocal microscopy assay.

**Figure 7:**
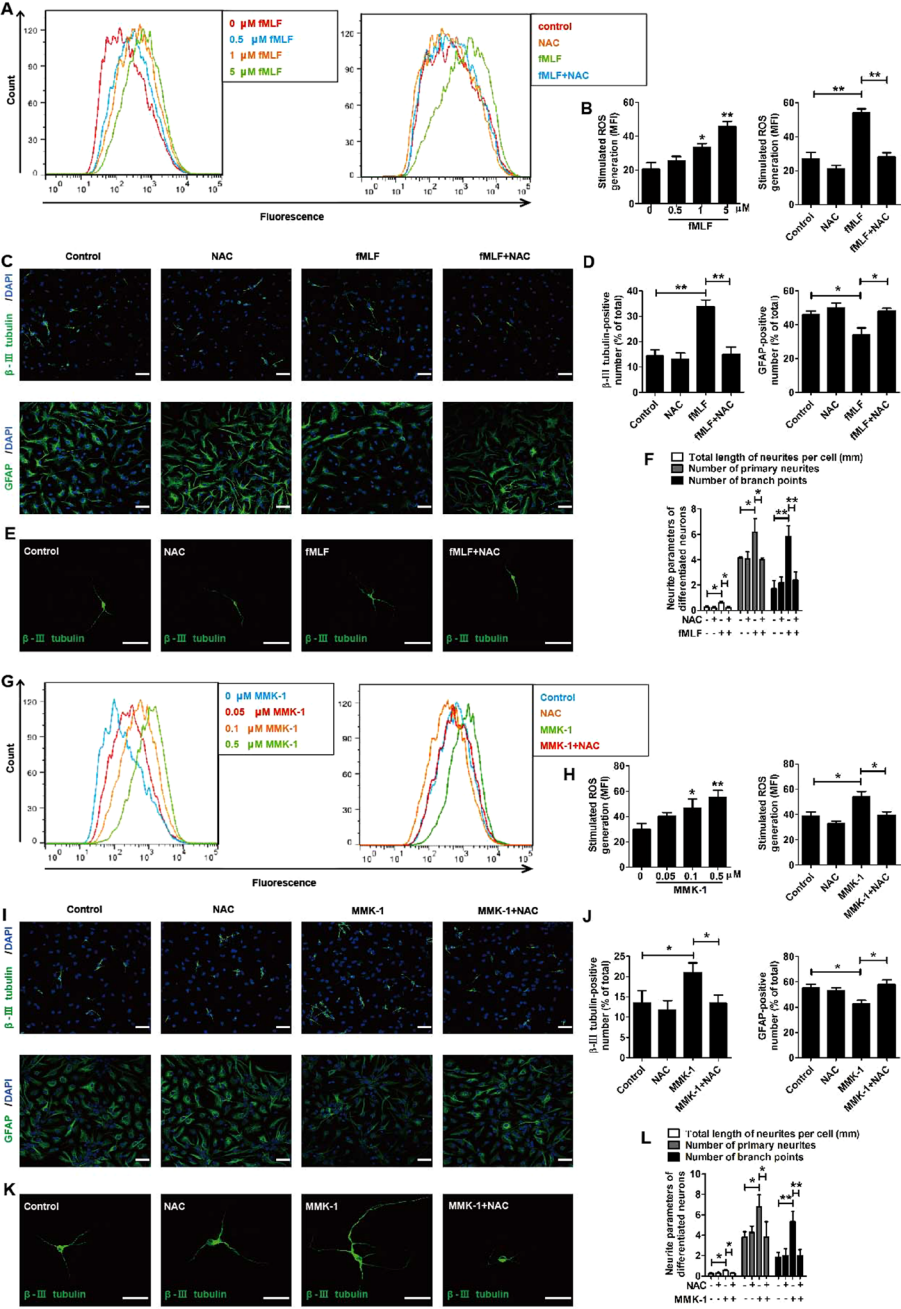
FPRs-induced neuronal differentiation of NSCs was mediated by ROS generation. (**A–F**) FPR1-induced neuronal differentiation of NSCs was mediated by ROS generation. (**A,B**) Flow cytometric analysis for generation of ROS by NSCs incubated in 0, 0.5, 1, and 5 µM fMLF for 6 h with or without preadministering NAC at 5 mM. (**A**) Representative plots of FPR1-mediated ROS generation. (**B**) Histograms for quantification of ROS generation. The levels of ROS were represented by the median fluorescence intensity. (**C,D**) Dissociated NSCs were differentiated with or without preadministering NAC for 30 min before administering fMLF. The images in (**C**) are representative of several independent experiments, and the graph in (**D**) shows the statistical results. (**E**) Dissociated NSCs were differentiated in an extremely low density (1000 cells/mL), and then the cells were immunostained with β-III tubulin. (**F**) Quantified analysis of the total length of neurites per cell, the number of primary neurites, and the number of branch points. (**G–L**) FPR2-induced neuronal differentiation of NSCs was mediated by ROS generation. (**G,H**) Flow cytometric analysis for generation of ROS by NSCs incubated in 0, 0.05, 0.1, and 0.5 µM MMK-1 for 6 h with or without preadministering NAC at 5 mM. (**G**) Representative plots of FPR1-mediated ROS generation. (**H**) Histograms for quantification of ROS generation. (**I,J**) Dissociated NSCs were differentiated with or without preadministering NAC for 30 min before administering MMK-1. The images in (**I**) are representative of several independent experiments, and the graph in (**J**) shows the statistical results. (**K**) Representative micrographs of the differentiated cells immunostained with β-III tubulin. (**L**) Quantified analysis of the total length of neurites per cell, the number of primary neurites, and the number of branch points. Data were presented as mean ± SEM; *P < 0.05; **P < 0.01; *N* = 4. Scale bar = 20 µm.

The flow cytometry results suggested that ROS was significantly increased in a concentration-dependent manner when NSCs were cultured with fMLF or MMK-1. Meanwhile, the effects of fMLF or MMK-1 were blocked by antioxidant NAC (Fig. 7A,B,G and H). The results of confocal microscopy indicated that the promoting effects of fMLF or MMK-1 on the differentiation of NSCs into neurons were blocked by NAC. Meanwhile, the inhibitive effects of fMLF or MMK-1 in the differentiation of NSCs into astrocytes were also blocked by NAC (Fig. 7C,D,I and J). Notably, NAC also eliminated the effects of fMLF or MMK-1 on the neurite outgrowth, the number of primary neurites, and the number of branch points of β-III tubulin−positive cells (Fig. 7E,F,K and L).

### FPRs-induced neuronal differentiation of NSCs was mediated by the PI3K-AKT signaling pathway

To further comprehend the mechanism through which fMLF or MMK-1 promoted neuronal differentiation of NSCs, the molecules associated with neuronal differentiation were assessed. As shown by previous reports from our laboratory, FPRs promoted the migration of rat NSCs^29^. Neutrophils and other motile cells respond to a chemoattractant gradient by rapidly adopting a polarized morphology^41^. AKT regulates the neutrophil polarization in response to fMLP^42^. Meanwhile NSCs have high endogenous ROS levels that regulate self-renewal and neurogenesis in a PI3K/Akt-dependant manner^43^. As shown by previous data, FPRs-induced neuronal differentiation of NSCs was mediated by ROS generation. The question is whether PI3K/Akt pathway is also a signaling molecule participating in the process during which FPRs strengthens NSCs’ potential to differentiate into neurons. The corresponding experiments were performed: the expression of β-III tubulin, GFAP and p-AKT/AKT was examined by Western blot; neuronal differentiation (percentage of β-III tubulin cells) and astrocyte differentiation (percentage of GFAP cells) were examined by confocal microscopy assay.

**Figure 8:**
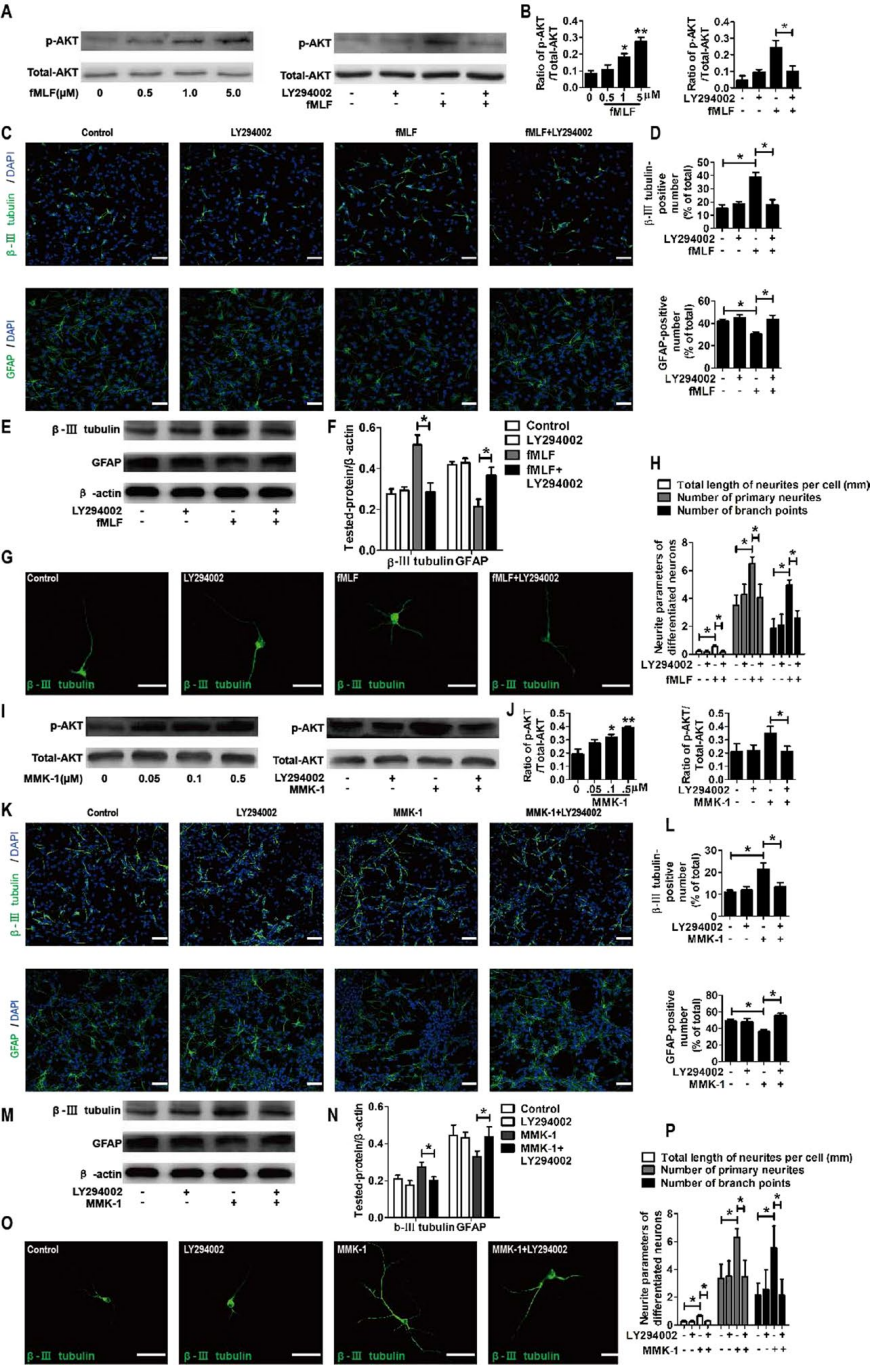
FPRs-induced neuronal differentiation of NSCs was mediated by the PI3K-AKT signaling pathway. (**A–H**) FPR1-induced neuronal differentiation of NSCs was mediated by the PI3K-AKT signaling pathway. (**A,B**) Western blot analysis of AKT activity in NSCs with or without preadministering LY294002 for 30 min before administering fMLF. (**A**) Representative immunoblots. (**B**) Relative quantification for AKT activity. (**C,D**) Dissociated NSCs were differentiated with or without preadministering LY294002 for 30 min before administering fMLF. The images in (**C**) are representative of several independent experiments, and the graph in (**D**) shows the statistical results. (**E**) Representative immunoblots. (**F**) Histograms showing relative levels of β-III tubulin and GFAP. (**G**) Dissociated NSCs were differentiated in an extremely low density (1000 cells/mL), and then the cells were immunostained with β-III tubulin. (**H**) Quantified analysis of the total length of neurites per cell, the number of primary neurites, and the number of branch points. (**I–P**) FPR2-induced neuronal differentiation of NSCs was mediated by the PI3K-AKT signaling pathway. (**I,J**) Western blot analysis of AKT activity in NSCs with or without preadministering LY294002 for 30 min before administering MMK-1. (**I**) Representative immunoblots. (**J**) Relative quantification for AKT activity. (**K,L**) Dissociated NSCs were differentiated with or without preadministering LY294002 for 30 min before administering MMK-1. The images in (**K**) are representative of several independent experiments, and the graph in (**L**) shows the statistical results. (**M**) Representative immunoblots. (**N**) Histograms showing relative levels of β-III tubulin and GFAP. (**O**) Representative micrographs of the differentiated cells immunostained with β-III tubulin. (**P**) Quantified analysis of the total length of neurites per cell, the number of primary neurites, and the number of branch points. Data were presented as mean ± SEM. *P < 0.05; **P < 0.01; *N* = 4. Scale bar = 20 µm.

The results of Western blot showed that p-AKT/AKT ratio significantly increased in a concentration-dependent manner when NSCs were cultured with fMLF or MMK-1. Meanwhile, the effects of fMLF or MMK-1 were blocked by a PI3K-AKT signaling pathway inhibitor LY294002 (Fig. 8A,B,I and J). The results of confocal microscopy indicated that the promoting effect of fMLF or MMK-1 on neuronal differentiation of NSCs could be blocked by a PI3K-AKT signaling pathway inhibitor LY294002. Meanwhile, the inhibitive effects of fMLF or MMK-1 on the differentiation of NSCs into astrocytes were also blocked by LY294002 (Fig. 8C,D,K and L). In addition, the expression of β-III tubulin and GFAP was examined by Western blot. The results showed that a significant increase in β-III tubulin levels and a significant decrease in GFAP levels induced by fMLF or MMK-1 were abolished by LY294002 (Fig. 8E,F,M and N). Notably, LY294002 also eliminated the effects of fMLF or MMK-1on the neurite outgrowth, the number of primary neurites, and the number of branch points of β-III tubulin-positive cells (Fig. 8G,H,O and P).

## Discussion

This study first observed an increased expression of FPR1 and FPR2 during the neuronal differentiation, and then found that the activation of FPRs promoted neuronal differentiation and inhibited differentiation of neural stem cells into astrocytes, while the newly generated neurons were with more primary neurites and branching points and longer neurites per cell. This study also found that FPR1 and FPR2 were expressed in both the neural stem cells (Lex^+^ and C1qR1^+^ cells) and the nonneural stem cells (Lex^−^ and C1qR1^−^ cells, mainly progenitor cells), and their expression levels did not differ significantly. Moreover, the flow cytometry–sorted Lex^+^ and C1qR1^+^ cells could produce more neurons and fewer astrocytes after a 7-day treatment with FPR agonists before differentiation. The aforementioned effects resulting from the activation of FPRs could be blocked by an antioxidant NAC and a PI3K-AKT signaling pathway inhibitor (LY294002).

As GPCRs, FPRs are also widely expressed in phagocytic cell types, such as neutrophils, macrophages, dendritic cells, and so forth^27, 44^. FPR ligands exist in the tissues of lesioned skin or livers infected by *Listeria monocytogenes* ^20, 21^. FPR ligands are derived either from bacterial proteins^45, 46^ and HIV-1^47^ or from endogenous mitochondrial proteins^48^, and is released as a result of cell death or severe dysfunction^49^. Under such conditions, neutrophils are induced to migrate to the lesions via activation of FPRs, which is important to the immune system. In recent years, FPRs have also been found in stem cells such as MSCs. FPRs expressed in MSCs promote the capability of MSCs to migrate and differentiate into osteoblasts^23–25^.

A previous study confirmed the expression of FPRs in the rat-derived NSCs and their promoting role in the migration of NSCs both *in vitro* and *in vivo* ^29^. The present study found that the expression of FPR1 mRNA, but not FPR2 mRNA, significantly increased in a time-dependent manner during differentiation of NSCs. However, results from Western blotting proved that the protein expression of FPR1 and FPR2 increased in the neural stem cells during the differentiation process. These results indicated that the expression of FPR1 and FPR2 was regulated from multiple levels, and the specific mechanisms require further investigations. Several studies^50–52^ have found that FPR2 was expressed in NSCs, but the expression of FPR1 in NSCs was not consistent. Wada *et al*. reported no expression of FPR1 in NSCs^50^. However, a few previous studies demonstrated the expression of FPR1 in NSCs^29^. Meanwhile, Several studies have found that FPR1 was expressed in neurons^28, 53, 54^. These works suggested that the expression of FPR1 was induced during neuronal differentiation of NSCs. The present study also showed that the activation of FPRs promoted the neuronal differentiation of NSCs, newly generating neurons with more primary neurites and branching points and longer neurites per cell. Meanwhile, it inhibits the differentiation of NSCs into astrocytes. The study by Wang also found that the activation of FPRs could induce neuronal differentiation in neural stem cells, but no inhibitory effect on differentiation into astrocytes was demonstrated. It was inferred that the reason for this deviation from the present study might be because of the use of cortex-derived neural stem cells from fetal mice with a different gestational age. The gestational age of neural stem cells used by Wang derived from the cortex of fetal mice was14.5 days, while that of neural cells used in the present study was 12.5 days. The younger cortex neural stem cells were reported to have a greater potential for neuronal differentiation^55^. Therefore, it was inferred that a greater number of neural stem cells had the potential for neuronal differentiation in the present study, which, in addition to the promoting effect of FPR activation in the neural stem cells for neuronal differentiation, magnified the impact on the capacity of neural stem cells to differentiate into astrocytes. Thus, FPRs promoted neuronal differentiation of neural stem cells while inhibiting their differentiation into astrocytes. This phenomenon may be of great significance to the potential use of NSCs as a therapeutic candidate for CNS injury.

Lex and C1qR1 are markers of neural stem cells. Lex^+^ and C1qR1^+^ cell clusters contain more neural stem cells compared with Lex^−^ and C1qR1^−^ cell clusters^32, 33^. This study found that cell clusters positive for Lex and C1qR produced more neurons and fewer astrocytes during differentiation after a 7-day treatment with FPR agonists, which may correlate with the fact that cell clusters positive for Lex and C1qR1 contain more neural stem cells than those negative for Lex and C1qR1. Therefore, in the present study, there may be more production of neuronal-restricted progenitors and fewer of glial-restricted progenitors during the activation of FPRs on NSCs, resulting in the production of more neurons and fewer astrocytes in the positive group after removing the stimulus factors. Of course, this inference requires more direct evidence to support. As mentioned earlier, cell clusters both positive and negative for Lex and C1qR1 express FPRs, showing no significant difference in the expression levels. Therefore, the activation of FPR should have effect on both of them theoretically. However, in the present study, based on the parameters, the effect of FPR on the negative cell cluster was not observed. In summary, the activation of FPRs induces neuronal differentiation and inhibits the differentiation of neural stem cells into astrocytes. A number of studies, similar to the present study, have also confirmed the expression of FPRs in neurons. Whether this is a correlation between the increased production of neurons and decreased production of astrocytes induced by the activation of FPRs, and whether FPRs are expressed in neurons require further investigations.

In a study conducted by He *et al*.^52^, binding of amyloid-b1–42 and FPR2 increased ROS generation in the adult hippocampal neural stem/progenitor cells. They showed that amyloid-b1–42 (Ab42) oligomer triggered senescent phenotype of neuronal stem/precursor cells (NSPCs) *in vitro*. Oligomerized Ab42 inhibited cell proliferation and differentiation. Their data suggested that Ab42 accelerated NSPC senescence via FPR2-dependent activation of its downstream ROS-p38 MAPK signaling, which limited the function of NSPCs and contributed to failure of neurogenesis. High levels of ROS can lead to lipid peroxidation, oxidative chain reactions, and damage to cellular macromolecules, leading to death of neural cells^56^. However, low, nontoxic levels of ROS seem to maintain the proliferative capacity of NSCs and modulate their differentiation potential^57–59^ In the present study, after activating FPR1 or FPR2 with fMLF or MMK-1, ROS generation in the NSCs increased, and the differentiation of NSCs into neurons was promoted. The present results induced by FPR2 were inconsistent with those of He *et al*. probably because of the differences in ligand and other experimental conditions. He *et al*. used Ab42 as the ligand, while the present study used MMK-1 as the ligand for FPR2. Different ligands may result in different biological effects. It was confirmed that FPR2 transduced anti-inflammatory or neuroprotective effects^60, 61^, but it could also mediate proinflammatory responses to serum amyloid A and other peptides^62–64^. The ability of FPR2 to mediate several biological effects may be traced to different receptor domains used by different agonists. Moreover, NSPCs isolated from the hippocampus of adult mice were adopted in the study by He *et al*., while cortex-derived neural stem cells of fetal mice were used in the present study, which might also contribute to the difference in the results of the two studies.

It was found that the activation of FPRs promoted the neuronal differentiation of NSCs and inhibited their differentiation into astrocytes, and this process was dependent on the PI3K-AKT signaling pathway. In many cells, the activation of FPRs can activate the PI3K-AKT signaling pathway, which plays a key role in exerting the biological effects of FPRs^65^. Accumulating evidence shows that some signaling pathways coupled to cytokines and chemokines are involved in NSC proliferation, migration, and differentiation. The PI3K-AKT signaling pathway alters NSC self-renewal and progenitor cell division and differentiation^66–68^. The PI3K-AKT pathways are required for neural differentiation of canine bone marrow stromal cells^69^ embryonic carcinoma cells, embryonic stem cells^70^, and PC12 cells^71^.

Collectively, this study reported the activation of FPRs promoted the differentiation of NSCs into neurons and inhibited their differentiation into astrocytes via increased ROS generation and AKT activation in NSCs.

## Methods

### Animals and ethics statement

All mice used in this study were purchased from experimental animal center of the Third Military Medical University (Chongqing). All animal procedures were performed according to the China’s animal welfare legislation for the protection of animals used for scientific purposes and approved by the local authorities of the Third Military Medical University for care and use of laboratory animals. All efforts were made to minimize the number of animals and decrease their suffering.

### Antibodies and reagents

All reagents and chemicals were purchased from Sigma−Aldrich (MO, USA), unless otherwise specified. Primary antibodies against glial fibrillary acidic protein (GFAP), Olig2, and β-actin were from Santa Cruz Biotechnology (CA, USA); SOX2, formyl peptide receptor 1 (FPR1) or 2(FPR2), beta-III tubulin, and Nestin were from Abcam Inc. (MA, USA); and AKT and p-AKT were from Cell Signaling Technology (MA, USA). Mouse C1qR1 PE-conjugated antibody and mouse Lex-1 PE or PerCP-conjugated antibody were from R&D System. Dulbecco’s modified Eagle medium:nutrient mixture F-12 (DMEM/F-12), fetal bovine serum (FBS), and B27 were from Gibco (CA, USA). Epidermal growth factor and fibroblast growth factor (FGF)-2 were from Peprotech (NJ, USA), and 0.25% trypsin−EDTA was from Hyclone (UT, USA).

### Primary neurosphere culture

NSCs were enzyme-dispersed from cortices of fetal E12.5 mouse pups as described previously^72^. Briefly, brains of pups were dissected and incubated in DMEM/F-12 containing 0.25% trypsin−EDTA and 250 U/mL DNase I at 37 °C for 30 min. Then, tissue pieces were washed twice in DMEM/F-12 with 10% FBS to inhibit the digestion of trypsin. The tissue samples were triturated using a fire-polished Pasteur pipette and passed through a 100-µm nylon cell strainer to harvest dissociated cell suspensions after washing twice with DMEM/F-12. Cortical cells were plated at a density of 250,000 cells/mL (density of ~65,000 cells/cm^2^) in a 100-mm culture dish and cultured in a DMEM/F12 medium supplemented with B27, 20 ng/mL EGF, and 20 ng/mL FGF-2 at 37 °C under humidified 5% CO_2_ conditions as recommended.

### Reverse transcription–quantitative polymerase chain reaction

The NSCs were plated at a density of 1 × 10^4^ cells/cm^2^ in six-well cell culture plates and grown in the medium containing 1% FBS for 6 days. Total RNA was extracted from cultured cells at days 0, 2, 4, and 6 using a TaKaRa MiniBEST Universal RNA Extraction Kit according to the manufacturer’s instructions (TaKaRa, Japan), and contaminating DNA was depleted with RNase-free DNase (Qiagen, CA, USA). Total RNA (2 µg) was reverse transcribed into cDNA using a PrimeScript II First Strand cDNA Synthesis Kit (TaKaRa, Japan), and an aliquot of cDNA mixture (0.2%) was used as polymerase chain reaction (PCR) template. Primers were as follows: Fpr1 (forward, 5′-CAT GAA CAA GTC TGC AGT GAA CCT-3′; reverse, 5′-AGG TTT ATG TCT ATT ACA GTA TAT-3′); Fpr2 (forward, 5′-TCT ACC ATC TCC AGA GTT CTG TGG-3′; reverse, 5′-TTA CAT CTA CCA CAA TGT GAA CTA-3′); glyceraldehyde-3-phosphate dehydrogenase (GAPDH) (forward, 5′-GGC CCC TCT GGA AAG CTG TG-3′; reverse, 5′-CCA GGC GGC ATG GCA GAT C-3′). The reaction was performed using a SYBR Green PCR Master Mix Kit in a Rotor-Gene 3000 (Corbett Research) with an initial denaturation step at 94 °C for 5 min, followed by 40 cycles each at 94 °C for 30 s, 54 °C for 20 s, and 72 °C for 30 s. GAPDH was used as an internal standard. Each experiment was performed in triplicate, and data from two batches of cultures were analyzed using Rotor-Gene version 4.6 (Corbett Research).

### Western blotting

Cultured cells after different treatments were homogenized with lysis buffer (Beyotime Biotechnology, Jiangsu, China). Proteins (10 µg per lane) were evaluated using a BCA Protein Assay Kit (Beyotime Biotechnology) and then separated by 10% sodium dodecyl sulfate polyacrylamide gel electrophoresis under reducing conditions and electroblotted onto polyvinylidene difluoride membranes (Roche, Basel, Switzerland). The membranes were blocked with 5% (w/v) nonfat dry milk in Tris-buffered saline with 0.1% (v/v) Tween 20 for 2 h at room temperature and subsequently incubated with primary antibody overnight at 4 °C with gentle agitation and horseradish peroxidase−conjugated secondary immunoglobulin Gs (1:5000) for 2 h at room temperature. All membranes were detected by a ChemiDoc + imaging system using Pierce Fast Western Blot Kits (Thermo Scientific, USA).

### Confocal microscopy

The neurospheres were dissociated into single cell by StemProAccutase Cell Dissociation Reagent and plated at a density of 1 × 10^4^ cells/cm^2^ in a confocal Petri dish and allowed to attach overnight. The cells were then grown in the medium containing different concentrations of fMLF (0–5 µM) or MMK-1 (0–0.5 µM) for 6 days. To investigate the FPRs- or ROS-mediated effects of fMLF or MMK-1, an FPR1 inhibitor tertiary-butyloxycarbonyl (tBOC) at 0.2 µM, FPR2 inhibitor WRW4 0.4 µM or antioxidant *N*-acetylcysteine (NAC) at 5 mM was added 30 min before administering fMLF or MMK-1. To evaluate the promoting effects of the phosphatidylinositol 3‐kinase (PI3K)-AKT signaling pathway on differentiation of NSCs along the neuron lineage of fMLF or MMK-1, a PI3K-AKT signaling pathway inhibitor LY294002 at 10 µM was added 30 min before administering fMLF or MMK-1. To better observe the length of neurites per cell, primary neurites, and branch points, the seeding density was adjusted to 500 cells/cm^2^ after digesting the neurospheres into single cells and seeded onto a polyornithine-coated confocal dish, such that the cells would spread out without accumulating into clusters after adherence. Other treatments were the same as the aforementioned methods. The cells were incubated in 2% paraformaldehyde in 0.01 M phosphate-buffered saline (PBS) (pH 7.4) for 15 min at room temperature, blocked with 5% bovine serum albumin and 0.3% v/v Triton X-100 in PBS, and incubated with primary antibody overnight at 4 °C and then with fluorescent secondary antibodies for 2 h at room temperature. 4′,6-Diamidino-2-phenylindole (DAPI) was used to counterstain nuclei for 5 min at room temperature. The images were obtained using a confocal microscope (Carl Zeiss LSM510, Germany) and examined using AxioVision4 software.

### Measurement of intracellular ROS

The intracellular ROS levels were measured as described previously^73^ using the flow cytometer. To measure intracellular reactive oxygen species (ROS) formation, the fluorescent probe dichlorodihydrofluorescein diacetate (DCFH-DA) was used. Briefly, the NSCs were plated at a density of 1 × 10^4^ cells/cm^2^ in six-well cell culture plates and allowed to attach overnight. The NSCs were incubated in 0, 0.5, 1, and 5 µM fMLF or 0, 0.05, 0.1 and 0.5 µM MMK-1 for 6 h with or without preadministering NAC at 5 mM. The cells were then washed twice with PBS and treated with 30 µM DCFH-DA for 30 min at 37 °C in 5% CO_2_. The level of ROS was analyzed using the flow cytometer at an excitation wavelength of 488 nm and an emission wavelength of 525 nm.

### Flow cytometric analysis and cell sorting

The neurospheres were dissociated into single cell by StemProAccutase Cell Dissociation Reagent. Neurosphere-dissociated cells doubly stained with C1qR1 or LeX and FPR1 or FPR2 antibody were assayed by flow cytometry. Briefly, the cells were resuspended in phosphate-buffered saline (PBS), blocked with 2% bovine serum albumin and stained with FPR1 or FPR2 antibodies for 1 h each at 4 °C in the dark. The cells were then washed in PBS and incubated on ice for 30 min with FITC-conjugated F(ab)2 fragments of goat anti-rabbit IgG. The cells were washed in PBS again and stained with mouse C1qR1 PE-conjugated antibody or mouse Lex-1 PE-conjugated antibody for 30 min each at 4 °C. After a further wash in PBS, cell surface fluorescence was analyzed immediately by flow cytometry. Isotype-matched mouse immunoglobulin served as controls.

Surface-labeled neurosphere-dissociated cells were analyzed, and different subpopulations were sort-purified using a FACSTAR^+^ flow cytometer. Briefly, neurosphere-dissociated cells were suspended in phosphate-buffered saline (PBS), blocked with 2% bovine serum albumin and stained with FPR1 or FPR2 antibodies for 1 h each at 4 °C in the dark. The cells were then washed in PBS and incubated on ice for 30 min with FITC-conjugated F(ab)2 fragments of goat anti-rabbit IgG. The cells were washed in PBS again and stained with mouse C1qR1 PE-conjugated antibody or/and mouse Lex-1 PE or PerCP-conjugated antibody for 30 min each at 4 °C. Isotype-matched mouse immunoglobulin served as controls. After three washes in PBS, the cells were sorted using a Becton Dickinson FACS Aria.

Cells were sorted with markers, including FPR1, FPR2, C1qR1 or/and LeX. Each cell population was sorted into a 6-well ultra-low attachment Plate. Cells were cultured in a DMEM/F12 medium supplemented with B27, 20 ng/mL EGF, and 20 ng/mL FGF-2, with or without preadministering tBOC for 30 min before administering fMLF or MMK-1. After 7 days, neurospheres were observed. The neurospheres were dissociated into single cell again. Neurosphere-dissociated cells were plated at a density of 1 × 10^4^ cells/cm^2^ in a confocal Petri dish and allowed to attach overnight. The cells were then grown in the medium containing 1% FBS for 6 days. The cells were stained with beta-III tubulin or GFAP antibody and assayed using a confocal microscope ascribed as above.

### Statistical analysis

All data were presented as mean ± SEM (standard error of mean), and statistical analyses were performed using GraphPad Prism v5.0 (GraphPad Software, Inc., USA). Statistical differences among test and control groups were analyzed by Student’s *t* test and one-way analysis of variance followed by Bonferroni’s multiple comparison post-test. A *P* value less than 0.05 was considered statistically significant.
